# Infantile restrictive cardiomyopathy: cTnI-R170G/W impair the interplay of sarcomeric proteins and the integrity of thin filaments

**DOI:** 10.1371/journal.pone.0229227

**Published:** 2020-03-17

**Authors:** Diana Cimiotti, Setsuko Fujita-Becker, Desirée Möhner, Natalia Smolina, Heidi Budde, Aline Wies, Lisa Morgenstern, Alexandra Gudkova, Thomas Sejersen, Gunnar Sjöberg, Andreas Mügge, Marc M. Nowaczyk, Peter Reusch, Gabriele Pfitzer, Robert Stehle, Rasmus R. Schröder, Hans G. Mannherz, Anna Kostareva, Kornelia Jaquet

**Affiliations:** 1 Department of Clinical Pharmacology and Molecular Cardiology, Ruhr-University of Bochum, Bochum, Germany; 2 Cardiology, Bergmannsheil and St. Josef Hospital, Clinics of the Ruhr-University Bochum, Bochum, Germany; 3 Cryoelectron Microscopy, BioQuant, Medical Faculty, University of Heidelberg, Heidelberg, Germany; 4 Vegetative Physiology, University of Cologne, Cologne, Germany; 5 Department of Molecular Biology and Genetics, Almazov Federal Medical Research Center, St. Petersburg, Russia; 6 Department of Women’s and Children’s Health and Center for Molecular Medicine, Karolinska Institute, Stockholm, Sweden; 7 Plant Biochemistry, Faculty of Biology and Biotechnology, Ruhr-University Bochum, Bochum, Germany; 8 Department of Anatomy and Embryology, Medical Faculty, Ruhr-University Bochum, Bochum, Germany; Heart and Diabetes Center NRW, UNiversity Hospital of the Ruhr-University Bochum, GERMANY

## Abstract

*TNNI3* encoding cTnI, the inhibitory subunit of the troponin complex, is the main target for mutations leading to restrictive cardiomyopathy (RCM). Here we investigate two cTnI-R170G/W amino acid replacements, identified in infantile RCM patients, which are located in the regulatory C-terminus of cTnI. The C-terminus is thought to modulate the function of the inhibitory region of cTnI. Both cTnI-R170G/W strongly enhanced the Ca^2+^-sensitivity of skinned fibres, as is typical for RCM-mutations. Both mutants strongly enhanced the affinity of troponin (cTn) to tropomyosin compared to wildtype cTn, whereas binding to actin was either strengthened (R170G) or weakened (R170W). Furthermore, the stability of reconstituted thin filaments was reduced as revealed by electron microscopy. Filaments containing R170G/W appeared wavy and showed breaks. Decoration of filaments with myosin subfragment S1 was normal in the presence of R170W, but was irregular with R170G. Surprisingly, both mutants did not affect the Ca^2+^-dependent activation of reconstituted cardiac thin filaments. In the presence of the N-terminal fragment of cardiac myosin binding protein C (cMyBPC-C0C2) cooperativity of thin filament activation was increased only when the filaments contained wildtype cTn. No effect was observed in the presence of cTn containing R170G/W. cMyBPC-C0C2 significantly reduced binding of wildtype troponin to actin/tropomyosin, but not of both mutant cTn. Moreover, we found a direct troponin/cMyBPC-C0C2 interaction using microscale thermophoresis and identified cTnI and cTnT, but not cTnC as binding partners for cMyBPC-C0C2. Only cTn containing cTnI-R170G showed a reduced affinity towards cMyBPC-C0C2. Our results suggest that the RCM cTnI variants R170G/W impair the communication between thin and thick filament proteins and destabilize thin filaments.

## Introduction

Cardiac troponin I (cTnI), the inhibitory subunit of the heterotrimeric troponin complex (cTn), plays a key role in inhibiting the actin-myosin interaction during diastole. Inhibition is abrogated during systole in response to the increase in cytosolic calcium ion (Ca^2+^) concentration and saturation of the Ca^2+^-binding subunit (cTnC) with Ca^2+^. This event leads to a series of conformational changes: the regulatory C-terminal region of cTnI is released from tropomyosin/actin, allowing after an azimuthal movement of tropomyosin the actin-myosin interaction. In addition, myosin binding protein C (cMyBPC), anchored to the thick filament, may also bind to actin with its N-terminus (domains C0-C2) to support the shift of tropomyosin to a more active state of the filament at low Ca^2+^ [1, 2]. The functional interplay of cMyBPC and troponin has been described before [3]. However, the molecular mechanisms are not yet fully elucidated. Furthermore, it is unknown how cardiomyopathy inducing mutations affect this interplay.

*TNNI3*, encoding cTnI, is the main target for mutations inducing restrictive cardiomyopathy (RCM), a rare disease with poor prognosis [4, 5]. RCM is characterized by an enhanced ventricular stiffness leading to a restrictive filling pattern during diastole and enlarged atria. Furthermore, myocardial hypertrophy or dilation is absent and left ventricle systolic function is preserved [6]. Restrictive cardiomyopathy can have many causes: metabolic disorders like diabetes, genetically caused storage diseases like amyloidosis, and point mutations of sarcomeric proteins inherited in a dominant autosomal manner (for review see also Muchtar et al. 2017 [7]). Point mutations have been identified in a number of different sarcomeric components such as the heavy myosin chain of β-myosin (*MYH7*), myosin binding protein C (*MYBPC3*), and the troponin subunits cTnC (*TNNC1*), cTnI (*TNNI3*) and cTnT (*TNNT2*) as well as in cytoskeletal components like filamin (*FLNC*), αB-crystallin (*CRYAB*), and desmin (*DES*) [3, 8–13]. A number of RCM causing point mutations were identified in cTnI (*TNNI3*) that are clustered in its C-terminal region [4, 14–17]. Most of these RCM mutations were shown to increase the Ca^2+^-sensitivity of the actin-myosin interaction [18–20], although the underlying molecular mechanisms may vary and are not well known.

Here we analyze at the protein level two different newly identified mutations in *TNNI3* leading to amino acid replacements at the same position in the regulatory C-terminus of cTnI, namely NP_000354.4:p.Arg170Gly and NP_000354.4:p.Arg170Trp (R170G and W). Both mutations led to a severe form of RCM in early childhood; the patients died within one year after diagnosis. Here we show that the interplay of sarcomeric proteins, i.e. thin and thick filament proteins, is altered dependent on the type of amino acid exchanged. Both replacements lead to a destabilization of reconstituted thin filaments that is partially reversed by cMyBPC.

## Materials and methods

If not otherwise indicated, all materials were purchased from Sigma Aldrich, Germany.

### Patient data and clinical examination

The study was performed as described by Kostareva et al. 2016 [8] according to Helsinki Declaration and approval was obtained from the local Ethics Committees in Almazov Medical Research Centre, St. Petersburg, Russia and Karolinska Institute, Stockholm, Sweden. Written consent was obtained from all patients and their representatives prior to investigation. The diagnosis of RCM was based on the WHO/International Society and Federation of Cardiology Task Force clinical criteria [6]. In pediatric patients the diagnosis was based on echocardiography features of RCM such as atrial dilation in combination with normal or nearly normal left ventricular size and preserved or nearly preserved systolic function (left ventricular end diastolic dimension z-score ≤3, left ventricular wall thickness z-score ≤3 and fractional shortening ≥0.25%) and cardiac characterization data.

### DNA preparation, sequencing and protein prediction analysis

Total genomic DNA was extracted from peripheral blood using FlexiGene DNA Kit (Qiagen) according to the manufacturer’s protocol. The *TNNI3* gene was screened for mutations using dye terminator sequencing. In both cases additional target sequencing including a panel of 108 cardiomyopathy- and channelopathy-associated genes was performed using Illumina MiSeq as previously described [8]. Prediction analysis was performed using MutationTester, PROVEAN, SIFT and MetaSVM prediction tools.

### Vector construction and mutagenesis

Human troponin I (*TNNI3*, NM_000363.5) cDNA was cloned into the pET3C vector as described before [21]. Site-directed mutagenesis was performed using polymerase chain reaction with primers containing the desired mutation (the sequences of all primers and restrictions sites are given in S1 Table). To obtain the cDNA of cardiac N-terminal Myosin binding protein C fragment (cMyBPC-C0C2) total RNA was isolated from human left ventricular tissue and reverse transcription was performed using the OneStep RT-PCR kit (Qiagen) with gene-specific primers flanking the cMyBPC domains C0-C2 (amino acids 1-452 according to the NP_000247.2 sequence). The cMyBPC-C0C2 cDNA was cloned into the pET28c vector in frame with the N-terminal His_6_-tag sequence. All plasmids were verified by sequencing.

### Protein expression and purification

Monomeric G-actin was isolated according to Pardee and Spudich [22] from acetone dried powder made from porcine or bovine heart muscle as well as from rabbit skeletal muscle and stored at -80°C in 5 μM triethanolamine, 0.5 mM adenosine triphosphate (ATP), 0.2 mM CaCl_2_, 0.3 mM NaN_3_, 5% (w/v) sucrose (pH 7.8). Actin filaments were obtained by polymerisation of G-actin at 100 mM KCl and 5 mM MgCl_2_ right before use.

Cardiac tropomyosin (Tpm) was chromatographically purified from acetone dried powder using a hydroxyapatite column according to Bailey et al. [23] and stored at -20°C in 0.2 M K_3_PO_4_, 1 M KCl, 2 mM dithiothreitol (DTT) (pH 7.0).

Cardiac myosin was isolated from fresh pig heart muscle by precipitation as described by Margossian and Lowey [24], myosin subfragment 1 (myosin S1) was obtained by enzymatic cleavage of myosin by papain. Myosin S1 was stored at -80°C in 20 mM K_3_PO_4_, 40 mM KCl, 2 mM MgCl_2_, 2 mM DTT (pH 6.5).

Human cardiac troponin C, T and I as well as the C0C2 fragment of myosin binding protein C were produced recombinantly in *E. coli* BL21(DE3). cTnC was isolated using DE52 cellulose as described by Babu et al. [25]. Isolation of cTnT was performed using DE52 cellulose according to Deng et al. [26]. Wildtype (WT) cTnI and the R170G/W variants were isolated using CM-Sepharose and CNBr-Sepharose previously coated with cTnC according to the manufacturer’s manual. Human cardiac troponin complexes containing cTnI WT or R170G/W were reconstituted from the individual subunits as previously described [26]. The subunits were mixed at an equimolar ratio under denaturing conditions (6 M urea, 0.5 M KCl) and reconstituted by slowly decreasing urea and KCl concentrations by stepwise dialysis. Residual monomeric and dimeric troponin subunits were removed by gel filtration using a Sephadex G75 column (2.5x45 cm). The troponin complexes were stored at -20°C in 50 mM Tris, 0.5 M NaCl, 5 mM KCl, 1 mM MgCl_2_, 2 mM DTT (pH 7.5).

The C0C2 fragment of cMyBPC, expressed with a N-terminal His_6_-tag, was purified using Ni^2+^-magnetic beads (Biotool/Selleckchem, Germany) according to the manufacturer’s protocol and stored at -80°C in 20 mM sodium phosphate, 0.5 M NaCl, 0.5 M imidazole (pH 7.4).

The purity and quality of all isolated proteins was checked using Laemmli sodium dodecyl sulfate polyacryl gel electrophoresis (SDS-PAGE; 12% gels) [27] and subsequent Coomassie staining.

### Skinned fibre preparation and troponin exchange

Skinned cardiac fibres were prepared from guinea pigs, 14-15 weeks of age, as approved by the local Animal Care and Use Committee. The endogenous troponin of the fibres was exchanged by the recombinant human troponin complexes as described previously for skinned murine cardiac fibres by Siedner et al. [28] except that the exchange buffer consisted of 10 mM Tris, 132 mM NaCl, 5 mM KCl, 1 mM MgCl_2_, 5 mM EGTA, 1 mM NaN_3_, 5 mM DTT, 0.5 mM AEBSF, 15 μM antipain, 0.8 μM aprotinin and 10 μM leupeptin (pH 7.0). The skinned fibres bundles were mounted horizontally between two clamps connected to a length driver and the tip of the force transducer. For the exchange, the fibres were incubated at RT for 3 h in the exchange buffer containing 3 mg/ml human troponin complex. The extent of troponin-exchange was quantified by SDS-PAGE (12.5% gels) and analysis of Coomassie stained gels using Phoretics.

### Force measurements

Force measurements of skinned papillary muscles from guinea pigs were performed as described for skinned murine fibres [28]. Maximal Ca^2+^‑activated force and Ca^2+^-sensitivity of isometric tension were examined by exposing the skinned fibres to activating solutions with defined free Ca^2+^-concentrations, ranging from pCa 7.0 to 4.7, until a plateau in force was reached.

### Surface plasmon resonance (SPR) spectroscopy

Interactions between troponin variants and G-actin, tropomyosin and cMyBPC-C0C2 were analyzed using Biacore 2000. Cardiac G-actin (from pig) and tropomyosin were covalently immobilized on CM5 sensor chips via amino coupling according to the manufacturer’s manual at a density of 150 RU to ensure response levels of troponin of about 100 RU (optimal for kinetic analyses). The association and dissociation rates were measured using troponin at concentrations between 0.01 and 10 μM.

### Ca^2+^-dependent activation of the thin filaments containing pyrene maleimide labeled tropomyosin

Tropomyosin was labeled using pyrene-maleimide (PM) as described by Graceffa and Lehrer [29]. Thin filaments were reconstituted from rabbit skeletal F-actin, PM-tropomyosin and recombinant troponin containing cTnI-WT or R170G/W at 4:1:1 molar ratio, respectively. Final protein concentrations in the assay were 0.25 μM actin, 0.06 μM PM-tropomyosin, 0.06 μM troponin and 0.05 μM myosin S1. Activation of thin filaments was measured via PM-Tpm excimer fluorescence at 340 nm (excitation) and 480 nm (emission) wavelengths at distinct free Ca^2+^-concentrations in presence of 1 mM ATP in black 96-well plates (total volume 100 μl) using an Infinite 200 microplate reader (Tecan). Fluorescence intensities were corrected for background fluorescence and subsequently normalized to F_pCa4.1_ = 1 and F_pCa9.4_ = 0. Data were fitted using the normalized Hill equation (SigmaPlot, Systat Software).

### Cosedimentation

Cardiac F-actin (from pig) was reconstituted with tropomyosin and troponin variants at a molar ratio of 7:1:1 with or without cMyBPC-C0C2 (equimolar to troponin). After centrifugation pellets and supernatants were analyzed via SDS-PAGE and densitometric analysis of the Coomassie stained 12% gels using Image Lab (Bio-Rad, Germany). The ratio of cTnI bound to the thin filament was calculated from band intensities of the pellet and corresponding supernatant.

### Microscale thermophoresis (MST)

Binding experiments were performed using Monolith NT.115 (NanoTemper Technologies) according to the manufacturer’s manual. For labeling with a fluorescent dye His_6_-tagged cMyBPC-C0C2 was incubated for 30 min with the Monolith His-Tag labeling Kit RED-tris-NTA (NanoTemper Technologies, Munich, Germany). Different amounts of the binding partners, cTn-WT, the cTn variants R170G and R170W, or individual cTn subunits (cTnC, cTnT, cTnI-WT, -R170G and -R170W) were mixed with the fluorescently labeled cMyBPC fragment and loaded into standard capillaries (NanoTemper Technologies). For the measurement the nanoRED channel was used. Data analysis was performed with the appropriate NTanalysis software (NanoTemper). For cTnC only a binding check was performed at 0 μM vs. 100 μM cTnC, while for all other binding partners also a dilution series was analyzed to determine K_D_ values by fitting the data to the Hill function according to the NTanalysis software.

### Electron microscopy

For imaging of actin filaments bovine cardiac [30] or rabbit skeletal muscle actin [31] was polymerized by addition of 2 mM MgCl_2_ and subsequently diluted to 0.1 mg/ml. The actin filaments were reconstituted with cardiac tropomyosin and cardiac troponin complexes containing either cTnI-WT or cTnI mutants at a molar ratio of 7:1:1 (actin:Tpm:cTn) in the presence or absence of cardiac myosin S1 and cMyBPC-C0C2. Reconstituted thin filaments or F-actin alone were placed on copper grids (400 mesh) with a porous carbon-coat and negatively stained with 1% uranyl acetate. The dried grids were examined in a Zeiss transmission electron microscope EM923 (SESAM) run at 150 kV fitted with a TemCamF416 camera (Tietz Video and Image Processing Systems, Gauting, Germany). Images were analyzed by counting filament breaks and kinks.

### Statistical analysis

Statistical analysis was performed using one way ANOVA and Dunnett’s post-test or unpaired Student’s t-test (GraphPad Prism). P-values<0.05 were considered to be statistically significant. If not otherwise indicated, data are given as mean ±standard error of the mean (SEM). Statistically significant differences were indicated as *(p<0.05), ** (p<0.01) and *** (p<0.001).

## Results

### Clinical characterization

Patient 1 showed an impaired left ventricular function and pulmonary hypertension at three years of age. He was diagnosed with RCM and died one year later of ventricular fibrillation. Heart biopsy showed mild cardiomyocyte hypertrophy and interstitial fibrosis. Upon genetic screening a missense mutation in *TNNI3* was found (chr:19:55665439:C>G; NM_000363.5:c.508 C>G) leading to a NP_000354.4:p.Arg170Gly (R170G) replacement in cTnI (corresponding chromatopherogram in S1A Fig). Patients 2 and 3, monozygotic twins, were diagnosed with RCM at the age of 8 and 13 months, respectively. Echocardiographic examination revealed a restrictive filling pattern and enlarged atria. Both children died at the age of 12 and 18 months, respectively, due to congestive heart failure and atrial fibrillation. A *TNNI3* mutation (chr:19:55665439:C>T; NM_000363.5:c.508 C>T) leading to a NP_000354.4:p.Arg170Trp (R170W) replacement in cTnI was found (corresponding chromatopherogram in S1B Fig) [8]. The involved amino acid Arg170 is conserved in mammals as well as in chicken, frogs, and even in Drosophila (S2 Fig). Target sequencing including a panel of 108 cardiomyopathy- and channelopathy-associated genes revealed to additional pathogenic or likely pathogenic variants in both cases. Both *TNNI3* variants were not found in gnomAD exome and gnomAD genome databases, but were reported in ClinVar in association with cardiomyopathy and revealed high probability of damaging effect using MutationTester, SIFT, PROVEAN and MetaSVM prediction tools. Thus, according to ACMG guidelines [32] R170W was classified as a pathogenic and R170G as a likely pathogenic variant. No familial history of cardiac disorders was reported in the R170W case, parental testing confirmed that the R170W variant occurred *de novo* (however, no parental proof was performed). Familial screening in the R170G proband documented a hypertrophic cardiomyopathy with restrictive filling pattern in patient’s father, but the family refused additional genetic testing.

To investigate the molecular effects of both mutations we analyzed in a first approach the force-Ca^2+^-relationship in skinned fibres.

### cTnI-R170G/W induce Ca^2+^-sensitization in skinned fibres

**Fig 1:**
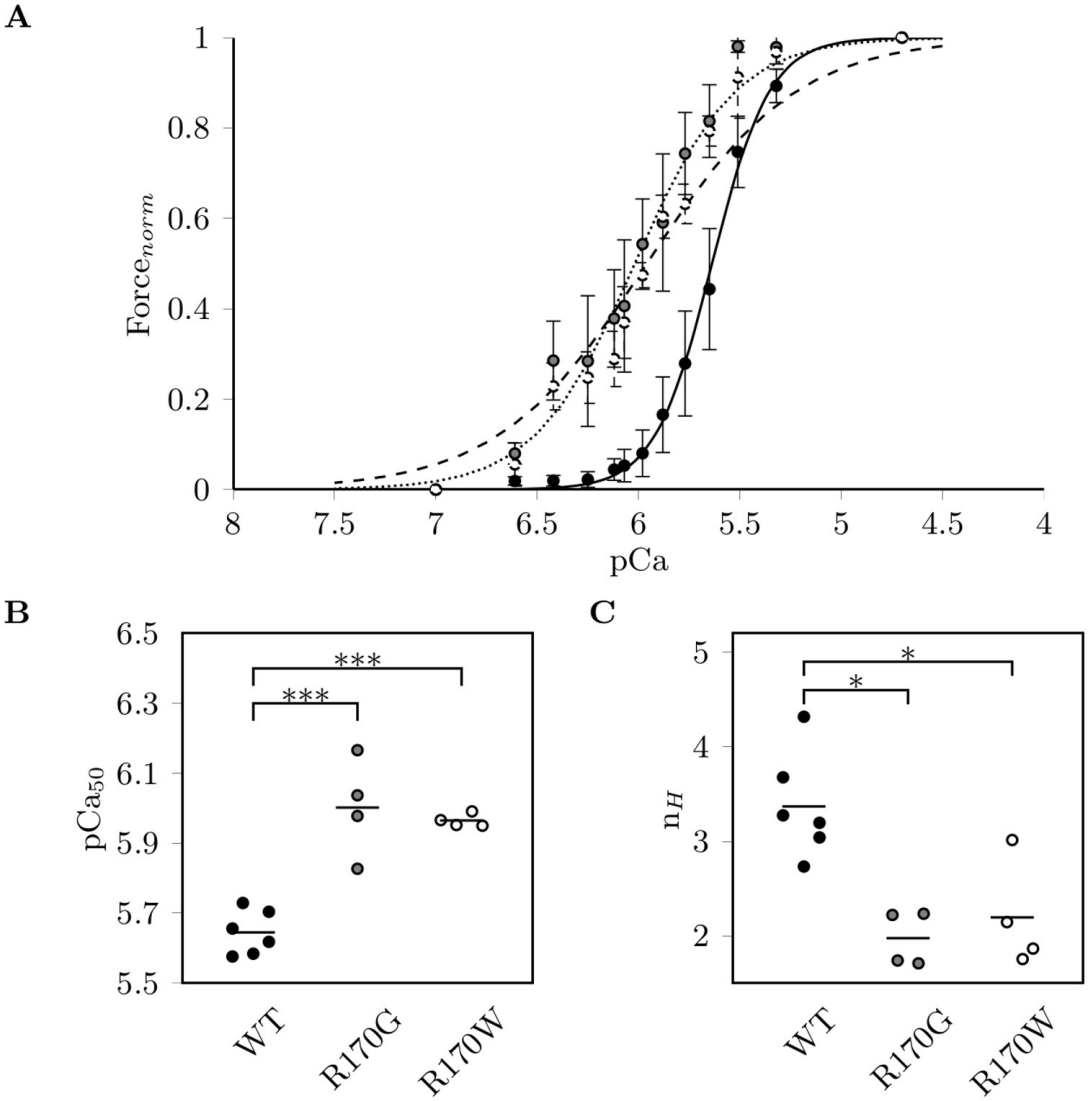
Ca^2+^-sensitivity of force development in guinea pig skinned fibres after exchange with Tn complexes. (A) Ca^2+^-dependent force development of guinea pig skinned fibres after exchange with troponin complexes containing cTnI wildtype (WT, black circles, solid line), R170G (grey circles, dotted line) or R170W (open circles, dashed line). Data are given as normalized force ±SEM (n = 6 for WT, n = 4 for R170G/W) vs. the negative logarithm of the Ca^2+^-concentration (pCa), fitted to the Hill equation. (B) Ca^2+^-sensitivity (pCa_50_) and (C) the cooperativity (Hill coefficient n_H_): horizontal bars indicate the mean of the individual measurements, * indicates statistical significance (ANOVA) with P<0.05; *** indicates statistical significance with P<0.001.

About 60% of the endogenous troponin in guinea pig skinned fibres was exchanged by recombinant human cTn containing the cTnI mutants or wildtype cTnI (a representative gel image of the replacement analysis is shown in S3 Fig). Force measurements were performed at distinct Ca^2+^-concentrations. While no significant differences in basal and maximal forces were found (S2 Table), both cTnI mutants showed an extremely large increase in Ca^2+^-sensitivity (pCa_50_) by about 0.3-0.4 pCa units compared to wildtype (Fig 1A and 1B, S3 Table). No significant difference was obtained between R170G and R170W. Furthermore, both mutants reduced the steepness of the force-pCa relation reflected by the lower Hill coefficient n_H_ (reduced by 41% for R170G and 35% for R170W, Fig 1C). The reduced cooperativity might reflect an impaired transfer of the Ca^2+^-signal, which could be due to disturbed protein-protein interactions.

### Interaction of cTn with actin and tropomyosin are affected differently by R170G/W

**Fig 2:**
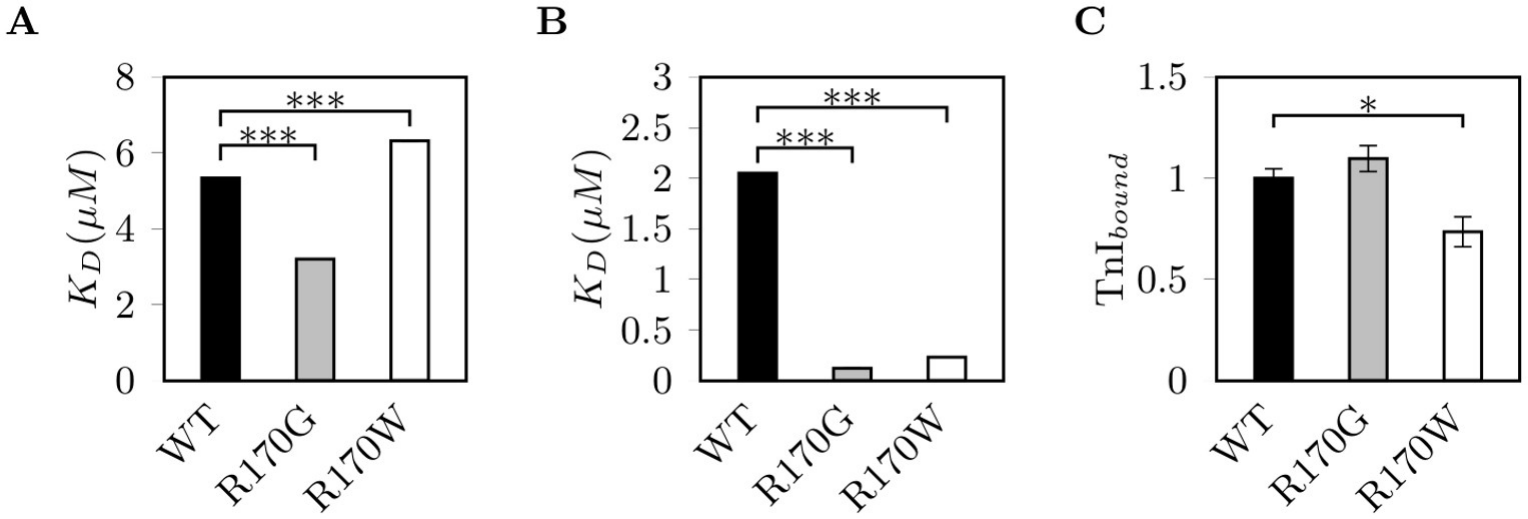
Interactions of cTn with actin, tropomyosin and reconstituted thin filaments. Binding kinetics of cTn containing cTnI-R170G/W (gray or white, respectively) or wildtype (WT, black) to immobilized (A) actin and (B) tropomyosin measured by SPR. Dissociation constants are given as K_D_ ±SEM (n = 3). (C) Binding of troponin containing cTnI-variants to F-actin decorated with tropomyosin analyzed by cosedimentation. Data given as amount of bound cTnI relative to total cTnI in the sample ±SEM (n = 20). * indicates statistical significance with P<0.05; *** indicates statistical significance with P<0.001.

We determined the kinetic parameters of the interaction between isolated porcine cardiac F-actin or tropomyosin with recombinant human troponin (cTn) containing cTnI-mutants or wildtype cTnI using surface plasmon resonance (SPR, Fig 2, S4 Table). cTn containing cTnI-R170G binds stronger to actin than wildtype cTn, the dissociation constant K_D_ was significantly reduced by 40% (5.34 ± (5 ⋅ 10^−4^) μM for WT vs. 3.20 ± (2 ⋅ 10^−6^) μM for R170G; Fig 2A). In contrast, cTn containing cTnI-R170W showed a significantly increased K_D_ (6.32 ± (2 ⋅ 10^−4^) μM) indicating a reduced binding to actin. Both mutants interacted stronger with tropomyosin, K_D_ was reduced by 80% (R170G) and 75% (R170W), respectively (2.05 ± (6 ⋅ 10^−5^) μM for WT vs. 0.12 ± (2 ⋅ 10^−4^) μM for R170G and 0.23±(6 ⋅ 10^−5^) μM for R170W; Fig 2B).

In addition, we analyzed the integration of cTn containing mutant or wildtype cTnI into reconstituted thin filaments by cosedimentation (Fig 2C, a representative gel image is shown in S4 Fig). Troponin containing cTnI-R170W integrated less sufficiently into the thin filament.

### Ca^2+^-dependent activation of reconstituted thin filaments

**Fig 3:**
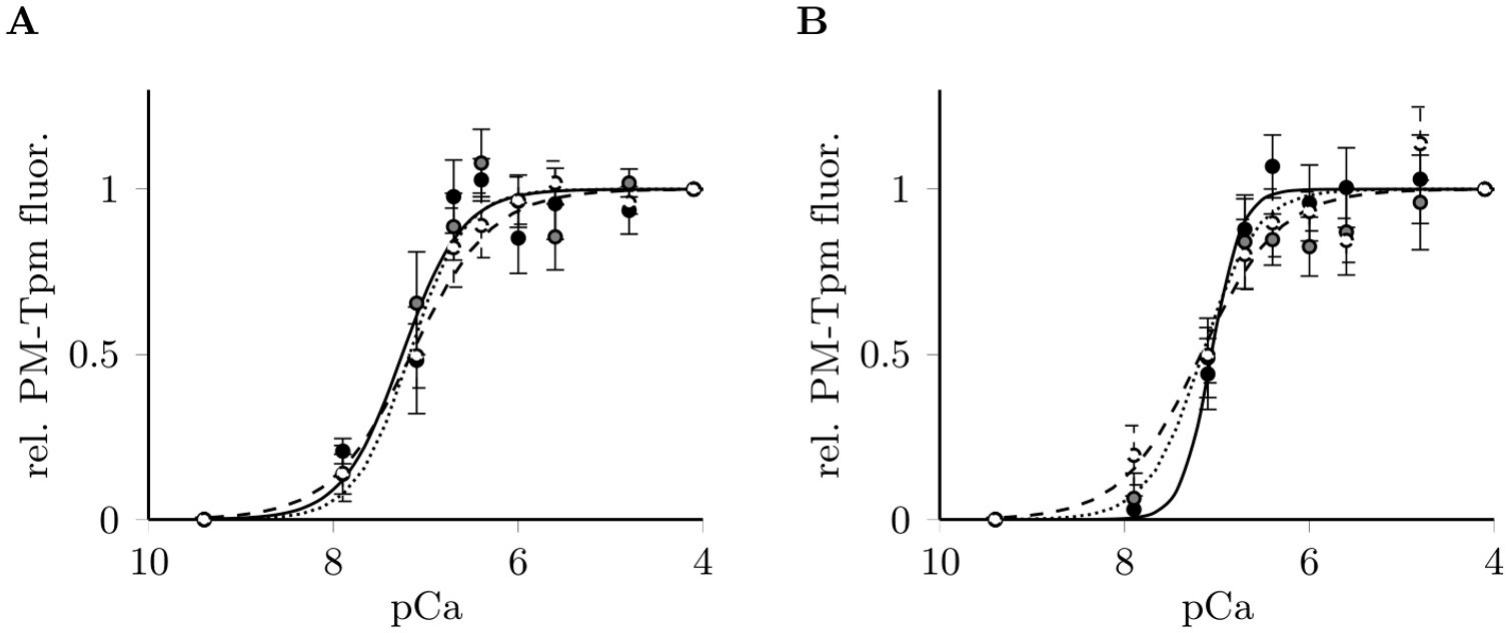
Ca^2+^-dependent activation of reconstituted thin filaments. Activation of thin filaments with troponin complexes containing cTnI variants: wildtype (black, solid line), R170G (gray, dotted line) and R170W (white, dashed line), measured by PM-tropomyosin fluorescence in presence of (A) myosin S1 and ATP as well as (B) cMyBPC-C0C2. Data are given as normalized fluorescence ±SEM (n = 5) vs. the negative logarithm of the Ca^2+^-concentration (pCa), fitted to the Hill equation to determine Ca^2+^-sensitivity (pCa_50_) and the cooperativity (Hill coefficient n_H_).

Using pyrene maleimide labeled tropomyosin (PM-Tpm) we measured the Ca^2+^-dependent activation of reconstituted cardiac thin filaments *in vitro* and calculated pCa_50_ and n_H_ in presence of myosin S1 and ATP. Surprisingly, cTnI-R170G/W did not show any significant effects on thin filament activation in this assay (Fig 3A, S5 Table). The stark contrast to the effects on Ca^2+^-sensitivity observed in skinned fibres indicates a possible involvement of further sarcomeric proteins present in native cardiac skinned fibres but absent in the *in-vitro*assay. An interesting candidate for such an involvement is cardiac myosin binding protein C (cMyBPC) which interacts with the thin filament and modulates Ca^2+^-sensitivity of contraction.

Addition of the N-terminal cMyBPC fragment C0C2 did not affect the Ca^2+^-sensitivity of thin filaments labeled with PM-Tpm. Instead, an increased cooperativity (n_H_) was observed for filaments containing cTnI wildtype, but not for R170G/W (Fig 3B). We concluded that cMyBPC-C0C2 affects the function of thin filaments differently in the presence of cTn containing cTnI wildtype or mutants. Based on these date we suspected a crosstalk between cMyBPC and troponin on the thin filament.

### cMyBPC-C0C2 affects cTn binding to reconstituted thin filaments and interacts directly with troponin

**Fig 4:**
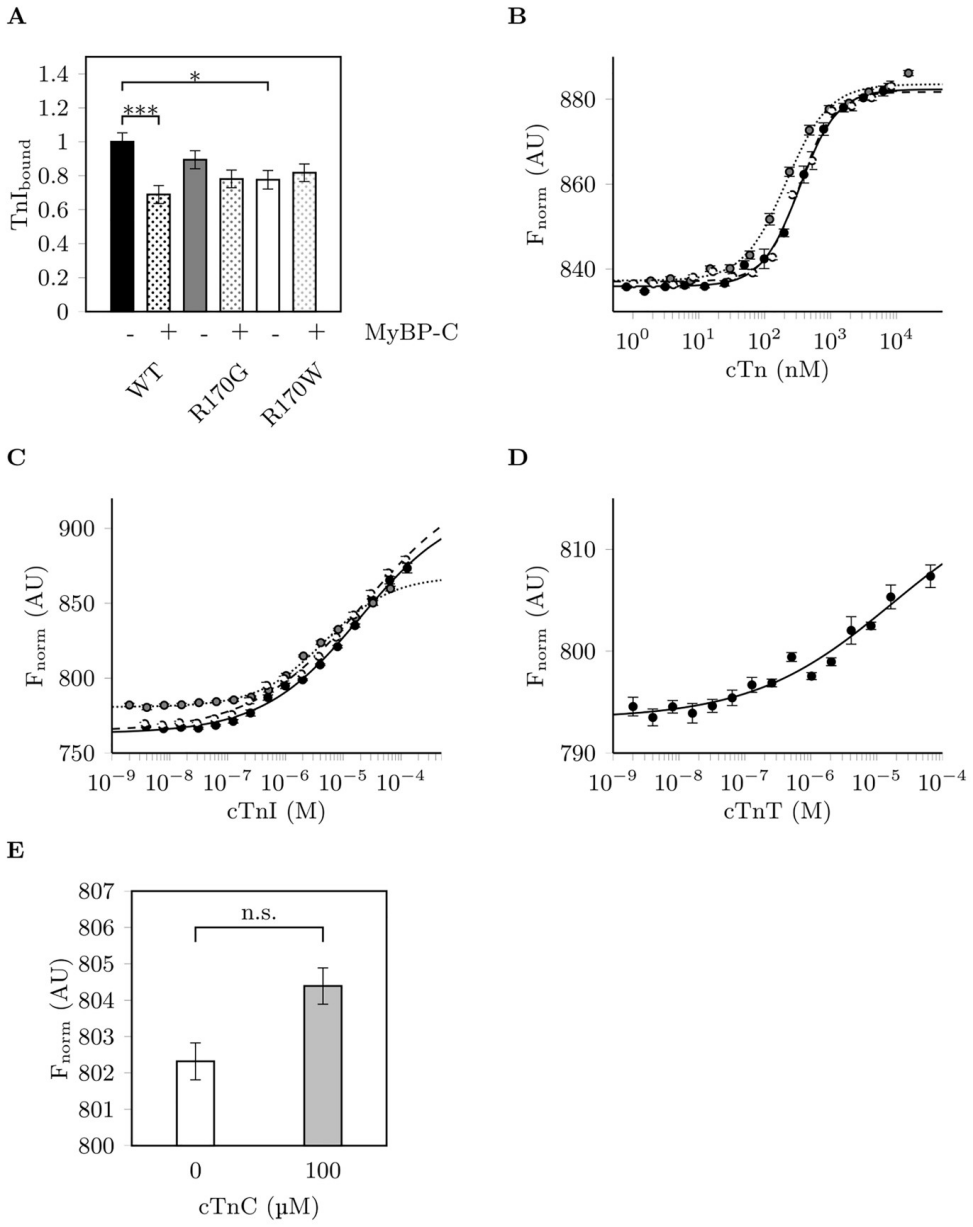
cMyBPC-C0C2 interacts with cTn and impacts its binding to thin filaments. (A) Binding of troponin containing wildtype (WT, black) cTnI or R170G/W (gray or white, respectively) to reconstituted thin filaments depending on the presence of cMyBPC-C0C2 analyzed by cosedimentation. Data are given as amount of bound cTnI relative to total cTnI in the sample ±SEM, normalized to wildtype cTnI in the absence of C0C2 (WT -C0C2, n = 15-18 each, * indicates statistical significance with P<0.05; *** indicates statistical significance with P<0.001). (B) Interaction of cMyBPC-C0C2 with troponin complexes containing cTnI-variants (mean normalized fluorescence ±SEM, AU: arbitrary units; wildtype: solid line, black circles; R170G: dotted line, gray circles; R170W: dashed line, white circles) as measured by MST. Data were fitted to the Hill function to determine EC_50_ (n = 5 for WT, n = 6 for R170G and R170W each). (C) Interaction of cMyBPC C0C2 with cTnI wildtype or R170G/W (as in panel B, n = 6 for WT, n = 5 for R170G and n = 7 for R170W). (D) Interaction of cMyBPC C0C2 with cTnT (mean ±SEM, n = 5). (E) No significant change in fluorescence (mean ±SEM, n = 4) and thus in cMyBPC thermophoresis was found in the presence of 100 μM cTnC, confirming the absence of an interaction.

The effects of cMyBPC-C0C2 on the binding of cTn variants to F-actin:tropomyosin were assessed in cosedimentation assays. While incorporation of cTn wildtype was significantly decreased after addition of C0C2, it was not changed for cTn containing cTnI-R170G/W (Fig 4A), indicating a disturbed response of cTnI mutants to the presence of cMyBPC. Furthermore, we found evidence for a direct interaction of cTn with cMyBPC-C0C2 using microscale thermophoresis (MST). cMyBPC-C0C2 was labeled via the N-terminal His_6_-tag with a NTA-fluorophore and thermophoresis was measured at different cTn concentrations (Fig 4B). While binding of cTn wildtype and R170W was comparable (K_D_ = 337±19 nM and 336±26 nM, respectively), the affinity of cTn containing cTnI-R170G towards cMyBPC-C0C2 was significantly increased (K_D_ = 211±15 nM, P = 0.002 vs. wildtype and R170W), probably affecting activation and stability of the thin filament.

Furthermore, using isolated troponin subunits instead of reconstituted troponin complexes, we found by MST that cMyBPC-C0C2 interacts with cTnI and cTnT, but not with cTnC (Fig 4C, 4D and 4E). However, the binding affinities towards cTnI and cTnT alone (K_D_ = 19.49±7.91 μM for cTnI wildtype and 17.1±3.6 μM for cTnT) were much lower than towards the troponin complex. Thus, we suggest that both cTnI and cTnT are required for an optimal interaction of cTn with cMyBPC-C0C2. In addition, no significant differences were found between the binding affinities of wildtype cTnI and cTnI R170G/W (K_D_ = 4.72±0.85 μM and 22.93±13.98 μM, respectively) towards cMyBPC-C0C2, which is in contrast to the results obtained with cTn complexes. Still, the general trend, that R170G shows a slightly higher affinity towards C0C2 than wildtype cTnI and R170W, remained unchanged.

### cTnI-R170G/W affect stability and structure of thin filaments

**Fig 5:**
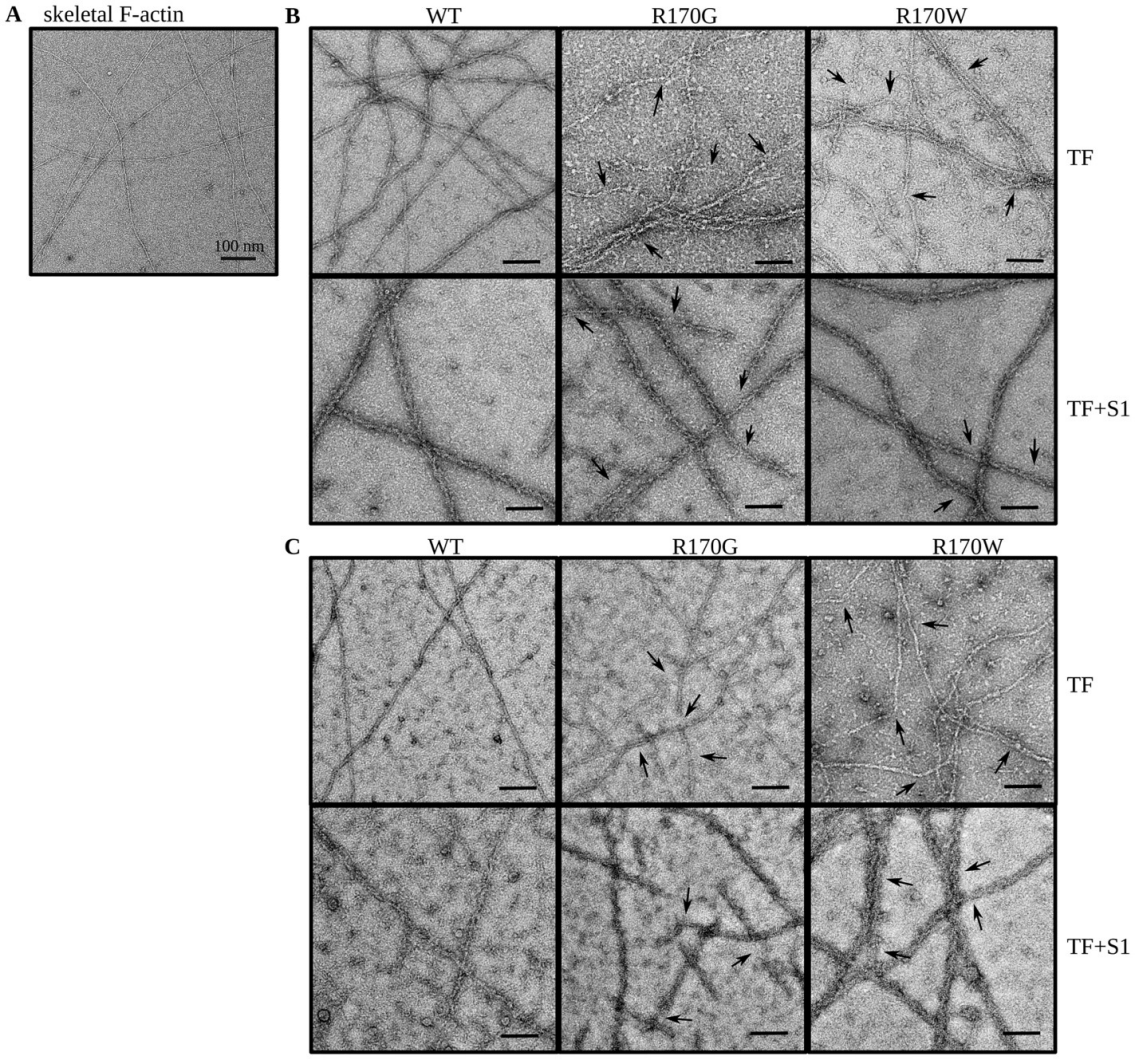
Stability and structure of thin filaments affected by cTnI mutants. Representative electron microscopic images of (A) rabbit skeletal F-actin, (B) reconstituted thin filaments (TF) from rabbit skeletal F-actin, porcine cardiac tropomyosin and human recombinant cardiac troponin complexes containing wildtype (WT) cTnI, R170G or R170W, and after decoration with porcine myosin S1 (TF+S1). (C) Thin filaments reconstituted in presence of human recombinant cMyBPC-C0C2. The black bars represent 100 nm in all images. The arrows indicate filament breaks, kinks and points of irregular decoration with myosin S1.

Thin filaments reconstituted from cardiac or skeletal actin, cardiac tropomyosin and cTn containing mutant or wildtype cTnI were analyzed by transmission electron microscopy after negative staining with uranyl acetate. In contrast to skeletal or cardiac actin filaments alone (Fig 5A and S5A Fig, respectively) a higher filament stability and linearity was obtained upon reconstitution of thin filaments containing cardiac tropomyosin and cTn-WT (Fig 5B). Only about 25% of filaments decorated with wildtype cTn were bent, whereas after decoration with cTnI-R170G about 40% of the filaments were bent and up to 40% were very short. With cTnI-R170W, about 60% of the filaments were bent and multiple breaks were found in 50%. In addition, bundling or aggregation of filaments was observed with cTnI-R170G/W. Since skeletal muscle filaments were more stable, images using skeletal muscle actin filaments are shown (Fig 5B). With cardiac actin filaments identical effects of cTn variants were observed (S5 Fig). Decoration with myosin subfragment 1 (rigor conditions) of both mutants straightened the filaments and reduced filament breaks.

After additional decoration with the N-terminal fragment C0-C2 of cMyBPC the number of bent filaments decreased to 37% and 41% for skeletal muscle F-actin decorated with Tpm and cTn containing cTnI-R170G and R170W, respectively, and the number of short filaments decreased to 20% for both cTnI variants (Fig 5C). Similarly, the number of filament breaks decreased by 50% for R170G, but only by 25% for R170W. Thus, the presence of cMyBPC-C0C2 appeared to improve the thin filament structure for cTnI-R170G/W, though full restoration was not observed.

## Discussion

RCM is a rare but severe form of cardiomyopathy with an early onset and high mortality. The underlying molecular pathomechanism is still debated, although it was soon recognized that RCM is a disease of the cardiomyocytes. Often missense mutations of a small number of sarcomeric proteins were made responsible for the high stiffness of the ventricular cardiomyocytes, although mutations in non-sarcomeric proteins like BLC2-associated athanogene 3 (*BAG3*), desmin and filamin have been described as well [3, 5, 7–9, 11, 13, 33]. Particularly common in RCM are point mutations within *TNNI3* encoding cTnI, which have been linked to the observed Ca^2+^-sensitization and the subsequent hypercontraction of the affected cardiomyocytes. Indeed, a number of single amino acid replacements concentrated mainly in the C-terminus of the cardiac troponin I subunit have been detected in RCM patients, all of them were reported to significantly increase ^2+^-sensitivity of force development [4, 14–17]. Though missense mutations of sarcomeric proteins are made responsible for cardiomyopathies, the molecular mechanism of the genotype to phenotype transition is in most cases not clearly evident and can vary depending on the individual mutation [10, 34]. A specific point mutation may lead to a cardiomyopathy phenotype at different lifespans, of different severity and furthermore a hypertrophic (HCM) phenotype may develop into a dilated (DCM) phenotype. Most of the previously characterized cTnI mutations, like R192H, K178E and and L144Q, linked to RCM show an onset in adolescence or adulthood, as well as a mixed phenotype with mild hypertrophy [4, 14]. This appears to be different in the cases reported here, in which the patients were heterozygous for a cTnI-R170G/W mutation leading to RCM in infancy. In humans the expression of the cardiac isoform of TnI is initiated shortly after birth, which subsequently substitutes the skeletal TnI isoform present during fetal life [35]. For cTnI-R170G/W, the disease onset probably correlates with the isoform shift to cardiac TnI. This suggests the existence of severe functional alterations or defects caused by these cTnI mutations. In order to understand the molecular basis of the effects of these mutations, we analyzed them by biochemical and physiological procedures in greater detail using purified wildtype cTnI and cTnI-R170G/W.

Using cardiac skinned fibres isolated from guinea pig it was possible to exchange about 60% of the endogenous troponin complex for human recombinant complexes containing mutated or wildtype cTnI. In these fibres the cTnI-R170G and R170W mutations enhanced the Ca^2+^-sensitivity of force generation, but reduced their cooperativity of force development, which is in agreement with the Ca^2+^-sensitization reported for other C-terminal cTnI mutations linked to RCM [15, 20, 36]. These data may well correlate with the heterozygous *in vivo* situation of the affected patients and show that the presence of the mutated cTnI to about 60% has a clear effect on the Ca^2+^-sensitivity of force generation.

Supposing that the effects of the mutated cTnIs will depend on altered interactions with other sarcomeric proteins, we further studied their interactions with actin, tropomyosin and myosin binding protein C. The residue R170 is located in the carboxy terminus of cTnI, which is thought to bind to actin/tropomyosin at low intracellular Ca^2+^-concentrations and to form the molecular switch together with the inhibitory region of cTnI [37]. The amino acids exchanges R170G/W lead to amino acids with completely different side chain properties. In the case of R170G, the arginine containing three non-polar methylene groups and a strongly basic guanidine group is replaced by the simplest amino acid glycine with only a hydrogen atom as side chain. This could enhance the conformational flexibility of the polypeptide backbone around the glycine residue. In the second mutation the arginine is replaced by the aromatic tryptophan with the largest side chain, a reactive indole. Thus, the local properties and the mode of cTnI interactions with other sarcomeric proteins might be considerably altered depending on the new residue.

Indeed, our data show that the affinity of troponin containing either cTnI-R170G or W to tropomyosin was clearly increased, possibly affecting cTn interaction with tropomyosin possibly at the Tpm-Tpm overlap region thereby leading to the decreased cooperativity of thin filament activation. The flexibility of the Tpm-Tpm overlap region is known to affect the cooperative activation of the thin filament [38].

In contrast, a distinctive feature between the R170G and R170W mutations was their different affinity to actin observed by SPR, as well as to actin filaments decorated with tropomyosin, which was decreased only for R170W as shown by cosedimentation assays. These data suggest an additional level of impairment of thin filament inhibition due to a possibly incomplete decoration by cTn. Thus, in thin filament activation assays we would expect an enhanced activity at low Ca^2+^-concentrations leading to a reduced amplitude. In fact, in enzyme coupled actin/myosin-ATPase activity assays we generally observed an increased basal activity and reduced amplitude with troponin containing cTnI-R170W, though the differences were not statistically significant (S6 Table). Additionally, the electron microscopic analysis revealed a considerable destabilization of thin filaments by R170W, which was also present but less prominent for R170G. Interestingly, decoration with myosin subfragment 1 improved thin filament stability for both mutants, though not to a level comparable with wildtype cTn.

Though cTn(R170G/W) enhanced Ca^2+^-sensitivity in skinned fibres, this was not observed in *in vitro* thin filament activation assays using PM-Tpm fluorescence. Such a discrepancy has been formerly described for HCM mutations [34] and might be due to the reduced system used in PM-Tpm assays being composed only of a few contractile and regulatory proteins in contrast to skinned muscle fibres. This implies that other sarcomeric proteins than cardiac troponin also contribute to Ca^2+^-sensitization. We found that the N-terminal cardiac myosin binding protein C fragment C0C2 has a pivotal effect on the cooperativity of Ca^2+^-dependent activation of thin filaments containing cTnI-WT, but not R170G/W. This might be due to altered interactions of C0C2 with the respective thin filament proteins. Indeed, in cosedimentation assays the overall incorporation of cTnI into reconstituted thin filaments in the presence of C0C2 was significantly decreased for wildtype cTnI but unchanged for R170G/W, indicating an altered response of R170G/W troponin to the binding of C0C2 to the thin filament.

Furthermore, we provide here for the first time evidence of a direct interaction between troponin and cMyBPC-C0C2, which was also affected by the mutations. While for cTn wildtype and R170W the binding affinity to C0C2 was similar, it was increased for R170G, again indicating an alteration of the interplay between the thin filament and cMyBPC by troponin containing cTnI-R170G. A further analysis using isolated cTn subunits revealed that the interaction between cMyBPC-C0C2 and troponin takes place via cTnI and cTnT, but not cTnC. Due to the low affinities found for the single subunits compared to the cTn complex, we suggest that both cTnI and cTnT contribute to the binding of cTn to C0C2 by forming a specific interaction site. In contrast to the cTn complexes, we could not determine significant binding differences between isolated wildtype and mutant cTnIs. This might be due to the general difficulty to perform the MST analysis at such high protein concentrations required to reach saturation (e.g. 20x K_D_) without inducing aggregation in the samples. Nevertheless, we found the same trend for cTnI-R170G showing an increased affinity to C0C2 as for the cTn complex containing R170G, supporting the idea of an altered interplay with C0C2 for this mutant. Though evidence for a cross-talk between cMyBPC and troponin has been reported before [3, 39], most of these studies analyzed the effects on phosphorylation levels, thus providing no mechanistic details about how this cross-talk takes place on the molecular level. Therefore, our findings introduce novel insights into a possible direct mechanism for a cMyBPC/cTn cross-talk. To what extent this interaction is affected by different phosphorylation states, is an interesting subject for future studies.

In support of a modulating effect of cMyBPC-C0C2 are EM data, which show that the thin filament structure was improved (i.e. straightened) after addition of C0C2 to filaments decorated with cTn containing cTnI-R170G/W. A similar effect was observed upon decoration with myosin-S1 emphasizing that the interplay with all the sarcomeric components is necessary to fully comprehend the effects of even a single amino acid replacement in a single sarcomeric component.

It is also known that the effects of HCM and DCM mutations on the Ca^2+^-sensitivity can be modulated by phosphorylation of sarcomeric proteins, especially of cTnI and cMyBPC [26, 40–43]. Though the overall phosphorylation background of sarcomeric proteins was not different in our skinned fibre experiments (S6 Fig, representative gel image in S7 Fig), we cannot exclude that some effects of R170G/W arise only in the presence of phosphorylated sarcomeric proteins. Additionally, even in the presence of cMyBPC-C0C2 the Ca^2+^-sensitivity of the thin filament activation obtained with R170G/W did not exceed the Ca^2+^-sensitivity of wildtype cTnI as seen in skinned fibre experiments. Therefore it is also conceivable that besides myosin binding protein C other proteins might contribute to the Ca^2+^-sensitization as considered typical for RCM. In fact, cTnI-R170G as well as R170W have previously also been reported in adolescent patients with HCM and RCM [44, 45]. The different onset of disease might be due to other factors as polymorphisms or mutations in proteins not analysed in these studies, as has been reported for a cMyBPC3 polymorphism in HCM [46].

An important source of differences between skinned fibres and *in vitro* measurements using reconstituted filaments is the amount of mutated cTnI employed. While we used only one cTnI variant in reconstituted filaments reflecting rather a monozygous situation, the exchange rate in skinned fibres was about 60%, thus there was still a significant amount of native guinea pig troponin present in the thin filaments. This can lead to dose dependent effects as observed by others analyzing mutations in myosin heavy chain or actin correlated with HCM [47–49]. The dose dependency might be an important factor in early childhood RCM. As the patients were heterozygous for these cTnI mutations, different expression levels of the mutated cTnI as well as different incorporation rates of the troponin complex into the sarcomere due to altered actin interaction are conceivable. Moreover, the predominant fetal TnI isoform is the skeletal TnI, which gradually is downregulated within the first eight months after birth, while cardiac TnI expression is upregulated [35]. Thus, there might occur a gradual increase of the amount of mutant cTnI leading to rising contractile dysfunction until pathogenic effects become prominent. As there was no cardiac tissue available from these patients, it was not possible to determine the expression levels of the mutated cTnI. Therefore, in this study we decided to analyze the effects of the mutations alone, though an analysis of mixtures of wildtype and mutant cTn is an interesting topic for future studies. In addition to dose dependent effects, a heterogeneity or cell-to-cell variability of mutant cTnI expression throughout the myocardium might trigger myofibrillar disarrays, distortions and arrhythmias, which cannot be adressed on the level of isolated proteins [49, 50].

A further limitation of the study is the usage of proteins isolated from different species: guinea pig skinned fibres, porcine and bovine cardiac actin, porcine cardiac tropomyosin, rabbit skeletal actin, as well as human recombinant troponin subunits and cMyBPC-C0C2. In mammals, the actin isoforms are tissue specific not species specific. Therefore human, bovine and porcine cardiac actin have identical amino acid sequences. This is not the case for tropomyosin, which might cause additional effects that would not occur in thin filaments containing human tropomyosin.

## Conclusion

Taken together, in case of cTnI-R170W the severe phenotype could be reflected in our *in vitro* experiments: diastolic, and probably systolic impairment, Ca^2+^ handling impairment, i.e. enhanced risk of malignant arrhythmia. Reduced dynamics due to stronger cTn—tropomyosin interaction as well as insufficient decoration of the thin filament due to weak actin interaction might contribute to enhanced stiffness of the contractile apparatus. For cTnI-R170G the effects on thin filament structure and interaction with actin were not as prominent as for R170W, which is again consistent with the later onset of disease. In this case, the stronger cTn—tropomyosin interaction as well as the altered interplay of the thin filament with cMyBPC seems to play a pivotal role. Although, involvement of other sarcomeric proteins might further affect sarcomere structure for cTnI-R170G/W in different ways. Still, it remains unclear why both mutants lead to a very similar phenotype despite of their very different side chain properties.

Our findings imply that altered interactions between sarcomeric proteins like cMyBPC, which directly interacts not only with actin, but also with troponin, contribute to the increase in Ca^2+^-sensitivity on sarcomere level. Thus, our data are of potential clinical importance, as substances altering Ca^2+^-sensitivity are often used to treat heart failure. The efficacy of these substances for patients carrying such mutations is not clear, though. Additionally, β-blockers affect cTnI and cMyBPC phosphorylation, which might alter their interaction in different ways in patients with cTnI mutations. Further studies have to clarify, whether this novel disease mechanism based on the impairment of complex interactions between cMyBPC and cTn is characteristic for severe RCM in early childhood or if it might also play a role in adolescent/adult RCM or other types of cardiomyopathies. Additional regulatory components such as effects of phosphorylation and dose-dependent effects have also to be taken into consideration.

## Supporting information

S1 Fig Electropherograms of the sequencing of *TNNI3* exon 7 of the patients’ DNA A: patient carrying the cTnI-R170G mutation (chr:19:55665439:C>G; NM_000363.5:c.508 C>G); B: patient carrying the cTnI-R170W mutation (chr:19:55665439:C>T; NM_000363.5:c.508 C>T).
(PDF)

S2 Fig Alignment of the cardiac troponin I sequence. Conserved amino acid residues in comparison to the human sequence are given as dots. The red box highligts the Arginin 170, which is conserved in all species down to Drosophila melanogaster.
(PDF)

S3 Fig Degree of replacement of endogenous (guinea pig) troponin by the recombinant (human) Tn-complex in fibres. After force measurements, fibres were fixed, homogenized and loaded on a prolonged SDS gel (12.5% AA). The extended run distance in combination with a longer and slower running time (5 mA, 45 V, 18 h) led to a greater resolution of proteins in the range of 25-40 kDa. Gel was stained with Coomassie Brilliant Blue R-250 and the relative amounts of the endogenous and recombinant TnI-band were determined by densitometry.
(PDF)

S4 Fig Representative gel image of a cosedimentation experiment with cTnI wildtype (WT) and R170G, stained with Coomassie. M is the protein standard (bands given in kDa), S is the supernatant, P the pellet of the respective sample. For data analysis, cTnI band densities were analyzed. In total, 10 gels were analyzed for each mutant, giving a total of n = 20 data points.
(PDF)

S5 Fig Representative electron microscopic images of cardiac F-actin (A), reconstituted thin filaments (TF) from cardiac F-actin, cardiac tropomyosin and cardiac troponin complexes containing wildtype cTnI (WT), cTnI-R170G or R170W (B). The black bars represent 100 nm in all images. The arrows indicate wavy filaments, filament breaks and kinks.
(PDF)

S6 Fig Phosphorylation analysis of guinea pig cardiac skinned fibres after exchange of troponin with recombinant human cardiac troponin containing cTnI wildtype (WT) or R170G/W. Fibres were treated with 15% TCA, homogenized and analyzed via SDS-PAGE with subsequent ProQ/SYPRO staining. Data are given as band intensity ratios of ProQ and SYPRO staining of the respective band ±SEM, n = 4.
(PDF)

S7 Fig Representative gel image of the protein phosphorylation analysis of skinned fibres from guinea pig after exchange with recombinant cTn complexes containing cTnI variants (WT, D127Y, R170G and R170W). Fibres were prepared from one 3-month old guinea pig (n = 1). Preparation of fibres and conditions for exchange were the same used for force measurements. For analysis a total number of 4 fibres were pooled. Note that the endogenous Tn is preserved in the control (ctrl) in which fibres were incubated in exchange buffer without the complex. Fibres were homogenized and separated on a 12.5% SDS gel. ProQ- and SYPRO-staining were performed according to manufactures instructions.
(PDF)

S1 Table Primers used for the site-directed mutagenesis of cTnI cDNA and for cloning of cMyBPC-C0C2 cDNA into pET28a. For the cTnI primers, the mutated position is bolded. For the cMyBPC primers, restriction sites included into the primer sequences are bolded and the corresponding enzyme indicated in brackets.
(PDF)

S2 Table Basal and maximal forces of guinea pig skinned fibres after exchange of the endogenous cTn to human recombinant cTn containing cTnI wildtype, R170G or R170W. F_pass_ is the basal or passive force and F_max_ the maximal force, all data normalized to the fibre cross section of the respective fibre, given as mean and standard deviation (SD). n is the number of fibres analyzed.
(PDF)

S3 Table Parameters of the force measurements of guinea pig skinned fibres after exchange of endogenous troponin to human recombinant troponin containing wildtype cTnI or cTnI-R170G or W at different Ca^2+^-concentrations. Forces were normalized to the fibre cross section of the respective fibre and the Ca^2+^-sensitivity pCa_50_ as well as the cooperativity n_H_ were calculated by non-linear regression using the Hill equation.
(PDF)

S4 Table Kinetic parameters of the interaction of troponin complexes containing wildtype cTnI (WT) or cTnI-R170G/W with actin and tropomyosin measured by surface plasmon resonance. k_on_ is the association rate constant, k_off_ is the dissociation rate constant, K_D_ is the dissociation constant. Data are given as mean ±SEM, n is the number of measurements.
(PDF)

S5 Table Parameters of the Ca^2+^-dependent activation of the thin filament, measured by pyrene maleimide labeled tropomyosin fluorescence. Ca^2+^- sensitivity is given as pCa_50_ ±SEM, cooperativity as the Hill coefficient n_H_ ±SEM, n is the number of measurements.
(PDF)

S6 Table Minimal activity and the activity amplitude of the actin/myosin S1-ATPase, measured in an enzyme coupled assay at pCa 9.8 and 4.5 as described before [26]. Briefly, reconstituted thin filaments containing wildtype cTnI, R170G or R170W were mixed with myosin S1 in the presence of cMyBPC. The ATP hydrolysis rate of actin/myosin S1 ATPase was measured by detecting inorganic phosphate release via an enzymatic cleavage of MESG by purine nucleoside phosphorylase. Data are given as activity ±SEM (normalized to the activity of unregulated F-actin). P values from Student’s t-test vs. cTnI-WT are given. n is the number of measurements.
(PDF)
