# Supplementary material for: Infantile restrictive cardiomyopathy: cTnI-R170G/W impair the interplay of sarcomeric proteins and the integrity of thin filaments
Source: PLoS One. 2020 Mar 17;15(3):e0229227. doi: 10.1371/journal.pone.0229227 (PMC7077804; doi:10.1371/journal.pone.0229227)
Supplement: S1 Fig — Electropherograms of the sequencing of TNNI3 exon 7 of the patients’ DNA A: patient carrying the cTnI-R170G mutation (chr:19:55665439:C>G; NM_000363.5:c.508 C>G); B: patient carrying the cTnI-R170W mutation (chr:19:55665439:C>T; NM_000363.5:c.508 C>T). (PDF) [file pone.0229227.s001.pdf]

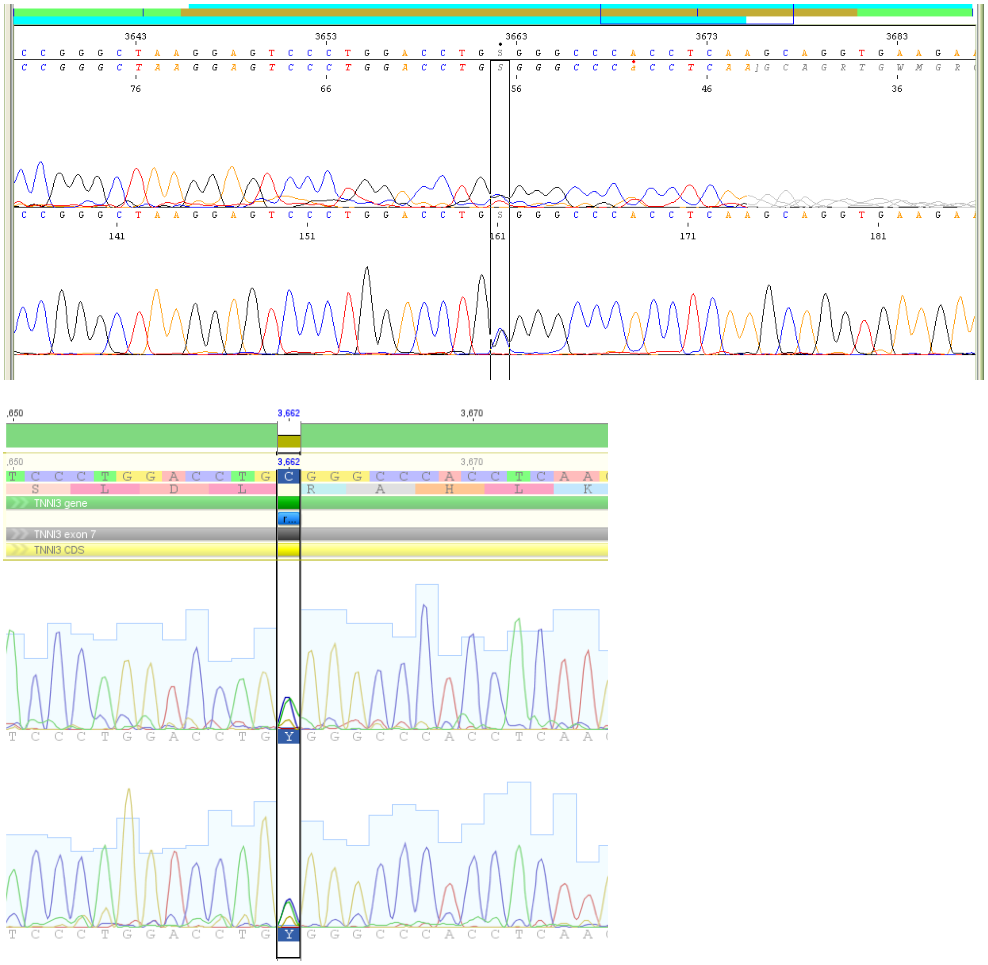

A

B

S1 Fig. Electropherograms of the sequencing of *TNNI3* exon 7 of the patients' DNA A: patient carrying the cTnI-R170G mutation (chr:19:55665439:C>G; NM_000363.5:c.508 C>G); B: patient carrying the cTnI-R170W mutation (chr:19:55665439:C>T; NM_000363.5:c.508 C>T).
