# Supplementary material for: Infantile restrictive cardiomyopathy: cTnI-R170G/W impair the interplay of sarcomeric proteins and the integrity of thin filaments
Source: PLoS One. 2020 Mar 17;15(3):e0229227. doi: 10.1371/journal.pone.0229227 (PMC7077804; doi:10.1371/journal.pone.0229227)
Supplement: S2 Fig — Conserved amino acid residues in comparison to the human sequence are given as dots. The red box highligts the Arginin 170, which is conserved in all species down to Drosophila melanogaster. (PDF) [file pone.0229227.s002.pdf]

```
Human   NP_000354.4     1    MADGSSDA-AREPRPAPAPIRRRSS-NYRAYATEPHAKKKSKISASRKLQLKTLLLQIAK  58
Rat     NP_058840.1     1    ...E....-.G..Q.....V.....A............................M.....  59
Bovine  NP_001035607.1  1    ...R.GGST.GDTV...P.V.....A............................M.....  60
Chicken AAH88784.1      44                       ......A...S.....QV.R.P...........S.M.....  84
Frog    NP_998735.1     14                        .KS.A-...G..V.....RQ.................R..  52
Fly     CAA42020.1      55                                                              .A  56

Human   NP_000354.4     59   QELEREAEERRGEKGRALSTRC-QPLELAGLGFAELQDLCRQLHARVDKVDEERYDIEAK  117
Rat     NP_058840.1     60   ..M.............V.....-...V.D....E......................V...  118
Bovine  NP_001035607.1  61   ......................-.................................V...  119
Chicken AAH88784.1      85   A.M...E...AR..E.Y.EEH.-E..Q.S..SLS.......E....I.V.......M...  143
Frog    NP_998735.1     53   R.....EQ..A...Q.H.GEL.-P.P..E...V.Q..E...E....IGR.......MGTR  111
Fly     CAA42020.1      57   E..KK.Q.RKAA.RR.IIEE..GS.RN.SDASEG...EI.EEYYE.MYICEGQKW.L.YE  116

Human   NP_000354.4     118  VTKNITEIADLTQKIFDLRGKFKRPTLRRVRISADAMMQALLGARAKESLDLRAHLKQV-  176
Rat     NP_058840.1     119  ...............Y...........................T...............-  177
Bovine  NP_001035607.1  120  ...........N....................................T..........-  178
Chicken AAH88784.1      144  .N...S..E..NL..........K.N.....L......R....TKH.V.M....N....K  203
Frog    NP_998735.1     112  .S..MA.MEE.RRRVAG--.R.V..A.....L......A....SKHRVGT....G.R..-  168
Fly     CAA42020.1      117  .R.KDW..N..NAQVN......VK.A.KK.SKYENKF--.K.QKK.A.-FNF.NQ..V.   172

Human   NP_000354.4     177  --KKEDTEKENREVGDWRKNIDALSGMEGRKKKFES  210
Rat     NP_058840.1     178  --....I............................   210
Bovine  NP_001035607.1  179  --.................................G  212
Chicken AAH88784.1      204  QT..D.AD.DI.........V..............   239
Frog    NP_998735.1     169  --R.D.A...S.........V..............A  202
```

S2 Fig. Alignment of the cardiac troponin I sequence. Conserved amino acid residues in comparison to the human sequence are given as dots. The red box highligts the Arginin 170, which is conserved in all species down to Drosophila melanogaster.
