# Supplementary material for: Infantile restrictive cardiomyopathy: cTnI-R170G/W impair the interplay of sarcomeric proteins and the integrity of thin filaments
Source: PLoS One. 2020 Mar 17;15(3):e0229227. doi: 10.1371/journal.pone.0229227 (PMC7077804; doi:10.1371/journal.pone.0229227)
Supplement: S3 Fig — After force measurements, fibres were fixed, homogenized and loaded on a prolonged SDS gel (12.5% AA). The extended run distance in combination with a longer and slower running time (5 mA, 45 V, 18 h) led to a greater resolution of proteins in the range of 25-40 kDa. Gel was stained with Coomassie Brilliant Blue R-250 and the relative amounts of the endogenous and recombinant TnI-band were determined by densitometry. (PDF) [file pone.0229227.s003.pdf]

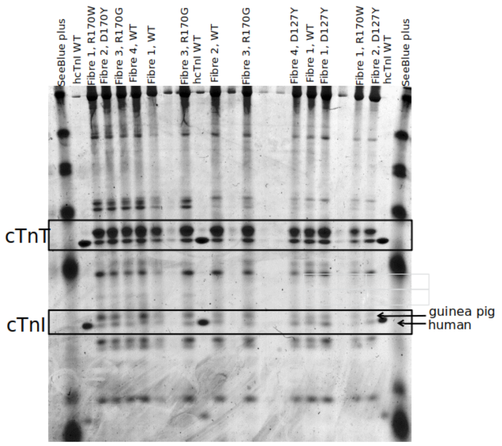

Complex	Exchange efficiency (%)
R170W	28
D127Y	28
R170G	45
WT	24
WT	26
R170G	33
WT	45
R170G	28
D127Y	55
WT	50
D127Y	57
R170W	37
D127Y	56

S3 Fig. Degree of replacement of endogenous (guinea pig) troponin by the recombinant (human) Tn-complex in fibres. After force measurements, fibres were fixed, homogenized and loaded on a prolonged SDS gel (12.5% AA). The extended run distance in combination with a longer and slower running time (5 mA, 45 V, 18 h) led to a greater resolution of proteins in the range of 25-40 kDa. Gel was stained with Coomassie Brilliant Blue R-250 and the relative amounts of the endogenous and recombinant TnI-band were determined by densitometry. The cTnI-D127Y variant shown on the gel was not included in this study.
