# Supplementary material for: Infantile restrictive cardiomyopathy: cTnI-R170G/W impair the interplay of sarcomeric proteins and the integrity of thin filaments
Source: PLoS One. 2020 Mar 17;15(3):e0229227. doi: 10.1371/journal.pone.0229227 (PMC7077804; doi:10.1371/journal.pone.0229227)
Supplement: S4 Fig — M is the protein standard (bands given in kDa), S is the supernatant, P the pellet of the respective sample. For data analysis, cTnI band densities were analyzed. In total, 10 gels were analyzed for each mutant, giving a total of n = 20 data points. (PDF) [file pone.0229227.s004.pdf]

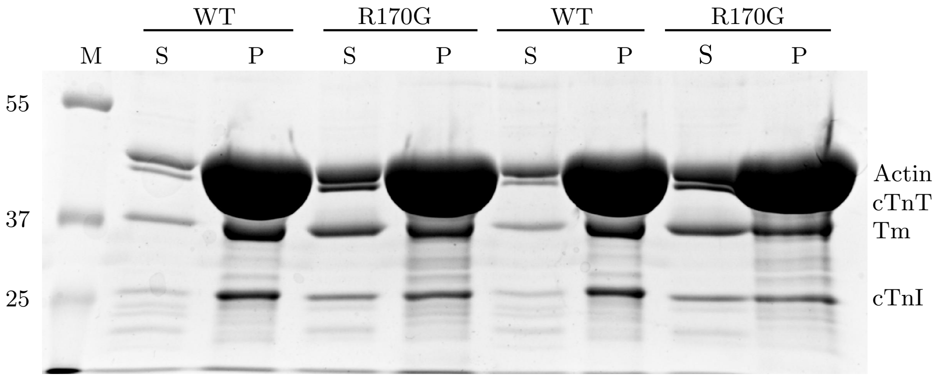

S4 Fig. Representative gel image of a cosedimentation experiment with cTnI wildtype (WT) and R170G, stained with Coomassie. M is the protein standard (bands given in kDa), S is the supernatant, P the pellet of the respective sample. For data analysis, cTnI band densities were analyzed. In total, 10 gels were analyzed for each mutant, giving an n=20 data points.
