# Supplementary material for: Infantile restrictive cardiomyopathy: cTnI-R170G/W impair the interplay of sarcomeric proteins and the integrity of thin filaments
Source: PLoS One. 2020 Mar 17;15(3):e0229227. doi: 10.1371/journal.pone.0229227 (PMC7077804; doi:10.1371/journal.pone.0229227)
Supplement: S5 Fig — The black bars represent 100 nm in all images. The arrows indicate wavy filaments, filament breaks and kinks. (PDF) [file pone.0229227.s005.pdf]

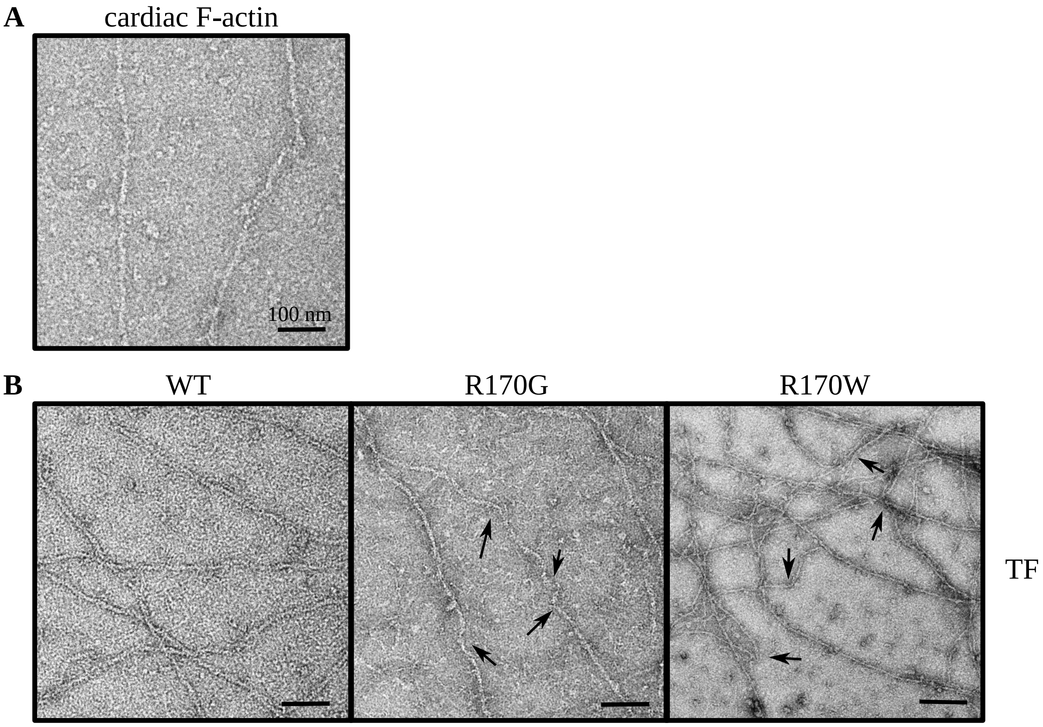

S5 Fig. Representative electron microscopic images of cardiac F-actin (A), reconstituted thin filaments (TF) from cardiac F-actin, cardiac tropomyosin and cardiac troponin complexes containing wildtype cTnI (WT), cTnI-R170G or R170W (B). The black bars represent 100 nm in all images. The arrows indicate wavy filaments, filament breaks and kinks.
