# Supplementary material for: Infantile restrictive cardiomyopathy: cTnI-R170G/W impair the interplay of sarcomeric proteins and the integrity of thin filaments
Source: PLoS One. 2020 Mar 17;15(3):e0229227. doi: 10.1371/journal.pone.0229227 (PMC7077804; doi:10.1371/journal.pone.0229227)
Supplement: S6 Fig — Fibres were treated with 15% TCA, homogenized and analyzed via SDS-PAGE with subsequent ProQ/SYPRO staining. Data are given as band intensity ratios of ProQ and SYPRO staining of the respective band ±SEM, n = 4. (PDF) [file pone.0229227.s006.pdf]

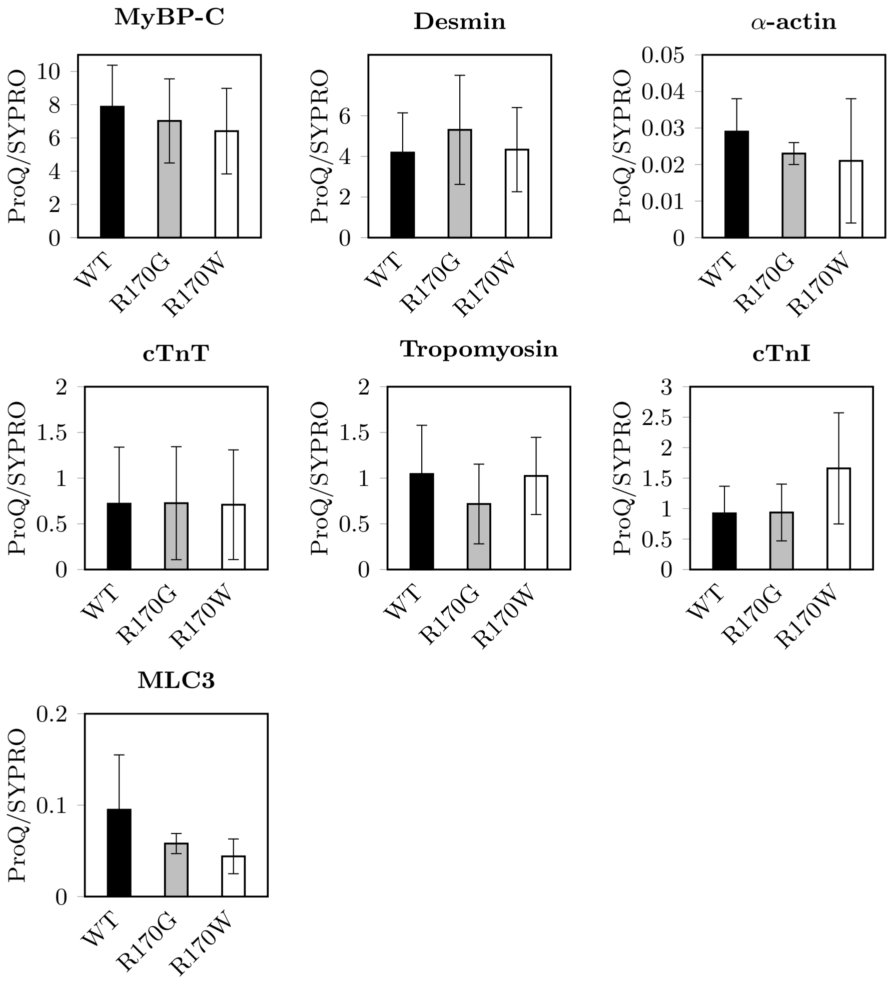

S6 Fig. Phosphorylation analysis of guinea pig cardiac skinned fibres after exchange of troponin with recombinant human cardiac troponin containing cTnI wildtype (WT) or R170G/W. Fibres were treated with 15% TCA, homogenized and analyzed via SDS-PAGE with subsequent ProQ/SYPRO staining. Data are given as band intensity ratios of ProQ and SYPRO staining of the respective band ±SEM, n=4.
