# Supplementary material for: Infantile restrictive cardiomyopathy: cTnI-R170G/W impair the interplay of sarcomeric proteins and the integrity of thin filaments
Source: PLoS One. 2020 Mar 17;15(3):e0229227. doi: 10.1371/journal.pone.0229227 (PMC7077804; doi:10.1371/journal.pone.0229227)
Supplement: S7 Fig — Fibres were prepared from one 3-month old guinea pig (n = 1). Preparation of fibres and conditions for exchange were the same used for force measurements. For analysis a total number of 4 fibres were pooled. Note that the endogenous Tn is preserved in the control (ctrl) in which fibres were incubated in exchange buffer without the complex. Fibres were homogenized and separated on a 12.5% SDS gel. ProQ- and SYPRO-staining were performed according to manufactures instructions. (PDF) [file pone.0229227.s007.pdf]

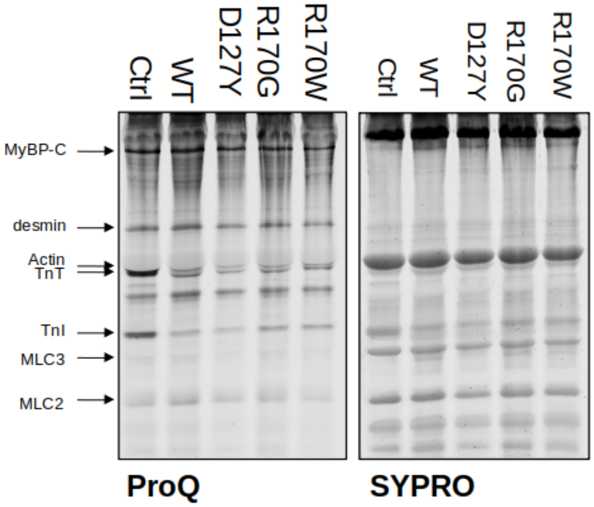

S7 Fig. Representative gel image of the protein phosphorylation analysis of skinned fibres from guinea pig after exchange with recombinant cTn complexes containing cTnI variants (WT, D127Y, R170G and R170W). The cTnI-D127Y variant was not included in this study. Fibres were prepared from one 3-month old guinea pig (n=1). Preparation of fibres and conditions for exchange were the same used for force measurements. For analysis a total number of 4 fibres were pooled. Note that the endogenous Tn is preserved in the control (ctrl) in which fibres were incubated in exchange buffer without the complex. Fibres were homogenized and separated on a 12.5% SDS gel. ProQ- and SYPRO-staining were performed according to manufactures instructions.
