# Supplementary material for: Infantile restrictive cardiomyopathy: cTnI-R170G/W impair the interplay of sarcomeric proteins and the integrity of thin filaments
Source: PLoS One. 2020 Mar 17;15(3):e0229227. doi: 10.1371/journal.pone.0229227 (PMC7077804; doi:10.1371/journal.pone.0229227)
Supplement: S1 Table — For the cTnI primers, the mutated position is bolded. For the cMyBPC primers, restriction sites included into the primer sequences are bolded and the corresponding enzyme indicated in brackets. (PDF) [file pone.0229227.s008.pdf]

S1 Table. Primers used for the site-directed mutagenesis of cTnI cDNA and for cloning of cMyBPC-C0C2 cDNA into pET28a For the cTnI primers, the mutated position is bolded. For the cMyBPC primers, restriction sites included into the primer sequences are bolded and the corresponding enzyme indicated in brackets.

cTnI-R170G (c.508 C>G)	for: 5'-GAGTCCCTGGACCTG**G**GGGCCCACCTCAAGC-3'<br>rev: 5'-GCTTGAGGTGGGCCC**C**CAGGTCCAGGGACTC-3'
cTnI-R170W (c.508 C>T)	for: 5'-GAGTCCCTGGACCTG**T**GGGCCCACCTCAAGC-3'<br>rev: 5'-GCTTGAGGTGGGCCC**AC**CAGGTCCAGGGACTC-3'
cMyBPC-C0C2	for: 5'-GGGAATTC**CATATG**ATGCCTGAGCCGGGGAAGAA-3' (NdeI)<br>rev: 5'-CTAG**GGATCC**CTAGGGCTCTTTCACAAAGA-3' (BamHI)
