# Supplementary material for: Infantile restrictive cardiomyopathy: cTnI-R170G/W impair the interplay of sarcomeric proteins and the integrity of thin filaments
Source: PLoS One. 2020 Mar 17;15(3):e0229227. doi: 10.1371/journal.pone.0229227 (PMC7077804; doi:10.1371/journal.pone.0229227)
Supplement: S2 Table — Fpass is the basal or passive force and Fmax the maximal force, all data normalized to the fibre cross section of the respective fibre, given as mean and standard deviation (SD). n is the number of fibres analyzed. (PDF) [file pone.0229227.s009.pdf]

S2 Table. Basal and maximal forces of puinea pig skinned fibres after exchange of endogenous troponin to human recombinant troponin containing wildtype cTnI or cTnI-R170G or W. F_{pass} is the basal or passive force and F_{max} the maximal force, both normalized to the fibre cross section of the respective fibre, given as mean and standard deviation (SD). n is the number of fibres analyzed.

	F_{pass} [mN/mm^2]	SD	F_{max} [mN/mm^2]	SD	n
**WT**	6.38	2.13	15.39	7.28	6
**R170G**	7.10	3.63	9.39	2.32	4
**R170W**	12.28	5.55	12.73	7.51	4
