# Supplementary material for: Infantile restrictive cardiomyopathy: cTnI-R170G/W impair the interplay of sarcomeric proteins and the integrity of thin filaments
Source: PLoS One. 2020 Mar 17;15(3):e0229227. doi: 10.1371/journal.pone.0229227 (PMC7077804; doi:10.1371/journal.pone.0229227)
Supplement: S3 Table — Forces were normalized to the fibre cross section of the respective fibre and the Ca2+-sensitivity pCa50 as well as the cooperativity nH were calculated by non-linear regression using the Hill equation. (PDF) [file pone.0229227.s010.pdf]

S3 Table. Parameters of the force measurements of guinea pig skinned fibres after exchange of endogenous troponin to human recombinant troponin containing wildtype cTnI or cTnI-R170G or W at different Ca^{2+}-concentrations. Forces were normalized to the fibre cross section of the respective fibre and the Ca^{2+}-sensitivity pCa_{50} as well as the cooperativity n_H were calculated by non-linear regression using the Hill equation.

	**WT**	**R170G**	**R170W**
**pCa_{50}**	5.618	5.827	5.952
	5.704	6.166	5.991
	5.584	6.037	5.95
	5.576	5.978	5.966
	5.729		
	5.656		
**pCa_{50} mean**	5.645	6.002	5.965
**n_H**	3.041	2.221	2.145
	3.196	1.739	1.754
	4.318	1.709	1.865
	3.677	2.234	3.014
**n_H mean**	3.369	1.975	2.195
