# Supplementary material for: Infantile restrictive cardiomyopathy: cTnI-R170G/W impair the interplay of sarcomeric proteins and the integrity of thin filaments
Source: PLoS One. 2020 Mar 17;15(3):e0229227. doi: 10.1371/journal.pone.0229227 (PMC7077804; doi:10.1371/journal.pone.0229227)
Supplement: S4 Table — kon is the association rate constant, koff is the dissociation rate constant, KD is the dissociation constant. Data are given as mean ±SEM, n is the number of measurements. (PDF) [file pone.0229227.s011.pdf]

**S4 Table. Kinetic parameters of the interaction of troponin complexes containing wildtype cTnI (WT) or cTnI-R170G/W with actin and tropomyosin measured by surface plasmon resonance.** $k_{on}$ is the association rate constant, $k_{off}$ is the dissociation rate constant, $K_D$ is the dissociation constant. Data are given as value ±SEM, n is the number of measurements.

| **Interaction** | | **WT** | **R170G** | **R170W** |
|---|---|---|---|---|
| **actin** | $k_{on}$ [$M^{-1}s^{-1}$] | $3880 \pm 3 \cdot 10^{-}1$ | $5430 \pm 2 \cdot 10^{-3}$ | $2610 \pm 6 \cdot 10^{-2}$ |
| | $k_{off}$ [$s^{-1}$] | $2.07 \cdot 10^{-2} \pm 1 \cdot 10^{-6}$ | $1.74 \cdot 10^{-2} \pm 7 \cdot 10^{-9}$ | $1.65 \cdot 10^{-2} \pm 4 \cdot 10^{-7}$ |
| | $K_D$ [µM] | $5.34 \pm 5 \cdot 10^{-4}$ | $3.20 \pm 2 \cdot 10^{-6}$ | $6.32 \pm 2 \cdot 10^{-4}$ |
| **tropomyosin** | $k_{on}$ [$M^{-1}s^{-1}$] | $1.58 \cdot 10^{7} \pm 4 \cdot 10^{2}$ | $2.86 \cdot 10^{7} \pm 2.1 \cdot 10^{2}$ | $7.1 \cdot 10^{6} \pm 8 \cdot 10^{2}$ |
| | $k_{off}$ [$s^{-1}$] [$s^{-1}$] | $32.4 \pm 3 \cdot 10^{-4}$ | $3.1 \pm 6 \cdot 10^{-3}$ | $1.63 \pm 4 \cdot 10^{-4}$ |
| | $K_D$ [µM] | $2.05 \pm 6 \cdot 10^{-5}$ | $0.12 \pm 2 \cdot 10^{-4}$ | $0.23 \pm 6 \cdot 10^{-5}$ |
| | n | 3 | 3 | 3 |
