# Supplementary material for: Infantile restrictive cardiomyopathy: cTnI-R170G/W impair the interplay of sarcomeric proteins and the integrity of thin filaments
Source: PLoS One. 2020 Mar 17;15(3):e0229227. doi: 10.1371/journal.pone.0229227 (PMC7077804; doi:10.1371/journal.pone.0229227)
Supplement: S5 Table — Ca2+- sensitivity is given as pCa50 ±SEM, cooperativity as the Hill coefficient nH ±SEM, n is the number of measurements. (PDF) [file pone.0229227.s012.pdf]

S5 Table. Parameters of the Ca^{2+}-dependent activation of the thin filament, measured by pyrene maleimide labeled tropomyosin fluorescence. Ca^{2+} sensitivity is given as pCa_{50} ±SEM, cooperativity as the Hill coefficient n_H±SEM, n is the number of measurements.

-cMyBP-C

cTnI	pCa_{50}	n_H	n
WT	7.26±0.11	1.34±0.32	5
R170G	7.18±0.08	1.49±0.35	5
R170W	7.16±0.07	1.00±0.14	5

+cMyBP-C

cTnI	pCa_{50}	n_H	n
WT	7.06±0.05	2.70±1.03	5
R170G	7.16±0.09	1.49±0.39	5
R170W	7.18±0.10	1.02±0.19	5
