# Supplementary material for: Infantile restrictive cardiomyopathy: cTnI-R170G/W impair the interplay of sarcomeric proteins and the integrity of thin filaments
Source: PLoS One. 2020 Mar 17;15(3):e0229227. doi: 10.1371/journal.pone.0229227 (PMC7077804; doi:10.1371/journal.pone.0229227)
Supplement: S6 Table — Briefly, reconstituted thin filaments containing wildtype cTnI, R170G or R170W were mixed with myosin S1 in the presence of cMyBPC. The ATP hydrolysis rate of actin/myosin S1 ATPase was measured by detecting inorganic phosphate release via an enzymatic cleavage of MESG by purine nucleoside phosphorylase. Data are given as activity ±SEM (normalized to the activity of unregulated F-actin). P values from Student’s t-test vs. cTnI-WT are given. n is the number of measurements. (PDF) [file pone.0229227.s013.pdf]

S6 Table. Minimal activity and the activity amplitude of the actin/myosin S1-ATPase, measured in an enzyme coupled assay at pCa 9.8 and 4.5 as described before [14]. Briefly, reconstituted thin filaments containing wildtype cTnI, R170G or R170W were mixed with myosin S1 in presence of cMyBP-C. The ATP hydrolysis rate of actin/myosin S1 ATPase was measured by detecting inorganic phosphate release via an enzymatic cleavage of MESG by purine nucleoside phosphorylase. Data are given as activity ±SEM (normalized to the activity of unregulated F-actin). P values from Student's t-test vs. cTnI-WT are given. n is the number of measurements.

cTnI	y_{min}	P(y_{min}) vs. WT	Amp	P(Amp) vs. WT	n
WT	0.43±0.08	–	0.88±0.09	–	9
R170G	0.37±0.06	0.557	0.74±0.08	0.262	9
R170W	0.64±0.06	0.058	0.61±0.09	0.052	8
